# Application of machine learning in understanding atherosclerosis: Emerging insights

**DOI:** 10.1063/5.0028986

**Published:** 2021-02-16

**Authors:** Eric Munger, John W. Hickey, Amit K. Dey, Mohsin Saleet Jafri, Jason M. Kinser, Nehal N. Mehta

**Affiliations:** 1 National Heart, Lung, and Blood Institute, National Institutes of Health, Bethesda, Maryland 20892, USA; 2 George Mason University, Fairfax, Virginia 22030, USA; 3 Johns Hopkins University, Baltimore, Maryland 21208, USA; 4 Stanford University, Stanford, California 94306, USA

## Abstract

Biological processes are incredibly complex—integrating molecular signaling networks involved in multicellular communication and function, thus maintaining homeostasis. Dysfunction of these processes can result in the disruption of homeostasis, leading to the development of several disease processes including atherosclerosis. We have significantly advanced our understanding of bioprocesses in atherosclerosis, and in doing so, we are beginning to appreciate the complexities, intricacies, and heterogeneity atherosclerosi. We are also now better equipped to acquire, store, and process the vast amount of biological data needed to shed light on the biological circuitry involved. Such data can be analyzed within machine learning frameworks to better tease out such complex relationships. Indeed, there has been an increasing number of studies applying machine learning methods for patient risk stratification based on comorbidities, multi-modality image processing, and biomarker discovery pertaining to atherosclerotic plaque formation. Here, we focus on current applications of machine learning to provide insight into atherosclerotic plaque formation and better understand atherosclerotic plaque progression in patients with cardiovascular disease.

## INTRODUCTION

Cardiovascular disease (CVD) including heart attack and stroke is the leading cause of death worldwide and is usually preceded by accelerated atherosclerosis.^1^ Atherosclerotic plaque formation, development, and progression are complex and involve many factors including elevated cholesterol, heightened immune activity, smooth muscle cell proliferation, and endothelial dysfunction.^2^ As plaques in the vessel wall build and progress and become high-risk, they may rupture and thus the associated arteries may be occluded distally, leading to impaired flow of oxygen-rich blood to the heart muscle, brain, and other parts of the body.

With such an impact on our health, substantial resources have been allocated to study atherosclerosis in large cohorts across multiple clinical, cellular, and molecular modalities. Consequently, there are a growing number of clinical and experimental datasets available that include multiple variables and risk factors known to be associated with atherosclerosis as well as many additional variables whose impact on disease is not well established.^3^ Ideally, the analysis of such datasets should include the development of both statistical and machine learning (ML) models that have minimal statistical bias and accurately account for important prognostic factors. Together, these models can be used to accurately quantify inferences within the data and forecast condition states useful for advanced treatment decisions. In fact, in a recent study designed to assess the benefits of artery-stenting in patients with atherosclerotic renal artery stenosis, the rigorous statistical analysis concluded that, when focused on a composite end point, there was no significant distinction among the treatment regiments.^4^ However, in a follow-up study designed to compare ML methods using the same data, 4 of 5 methods tested showed a small but distinct ability to identify the patients that achieved the same end point.^5^ This critical review aims to highlight the successful analysis of well-characterized datasets through ML methods that are inherently capable of minimizing statistical bias with a focus on atherosclerotic plaque. First, we briefly discuss the complex process of atherogenesis. Then, we show how ML has been utilized in atherosclerosis and CVD research and then focus on a recent study exploring the role of ML in risk stratifying psoriasis patients at high risk of developing coronary artery disease.^6^ Finally, we include a discussion of potential biological insights and the future impact and direction in the application of ML for novel biomarker discovery in atherosclerosis.

### Biology of atherosclerosis

Atherosclerosis is one of the leading causes of mortality and morbidity in developed countries and is widely recognized as a chronic inflammatory disease in addition to being a lipid-associated disease.^2^ It is a complex disease involving many different immune cell types, cytokines, chemokines, hemodynamics, and biomechanics of the vessel walls. Atherosclerosis leads to the formation of plaques that thicken large and medium-sized arterial walls, narrowing the lumen, which obstructs blood flow.^2,7^ Foam cells within plaques activate platelets and endothelium cells, which induces the migration and accumulation of vascular smooth muscle cells (VSMCs) from the media to the intima (layers of the vessel wall). VSMCs proliferate within the intima and secrete extracellular matrix macromolecules.^8^ Subsequently, VSMCs and leukocytes are activated, which in turn increases inflammatory cell infiltration in the atherosclerotic lesion. This process is accompanied by the eventual apoptosis of VSMCs.^8^ The combination of greater inflammatory cell infiltration and apoptotic death together leading to hypoxia-induced necrosis promotes the development of the plaque into the late fibroatheromatous lesion with eventual neovascularization. ^9^ These nascent immature micro-blood vessels are inherently leaky and permit extravasation of erythrocytes into the plaque, further contributing to necrotic core enlargement. ^9^ The many accumulating cells, including macrophages and activated endothelial cells, promote pro-inflammatory cytokines, matrix metalloproteinases (MMPs), and cathepsins.^10^ This, along with Interferon-alpha inhibition of collagen formation by VSMC, weakens the already fragile fibrous cap, increasing the likelihood of rupture.^11^ This allows simple mechanical agitation from blood flow to cause the atherosclerotic cap covering the inflammation site to rupture, resulting in possible blood coagulation and thrombosis, which leads to decreased blood flow distally and thus a vascular event.

Atherosclerotic plaques may stabilize or reduce in size, lipid content, foam cell content, and macrophage inflammation through a process known as atherosclerosis regression.^12^ This process is driven primarily by the Macrophage Reverse Cholesterol Transport (RCT) mechanism, allowing for atherosclerotic plaques to eliminate cholesterol. Enhancing foam cell cholesterol efflux by high-density lipoprotein (HDL) particles is considered a primary activator of the RCT mechanism, which is believed to offer atheroprotection.^13^

### Machine learning and atherosclerosis

**FIG. 1.**
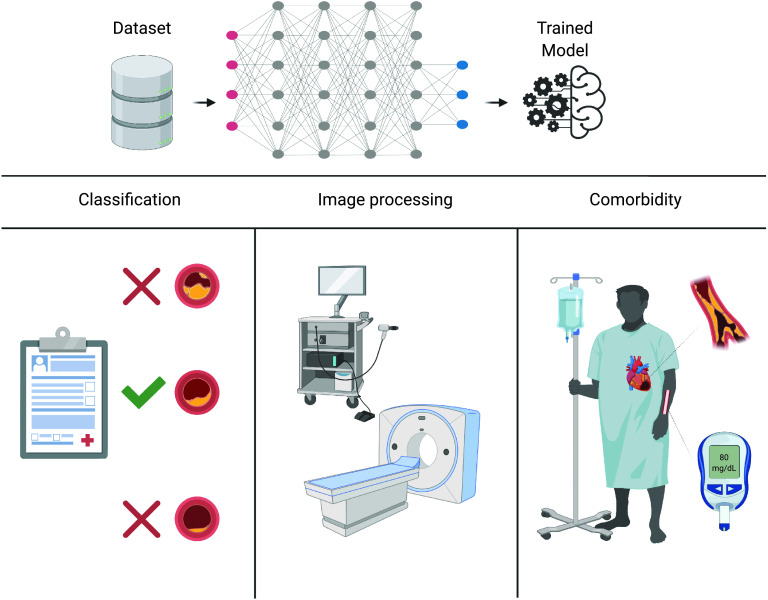
Machine learning modeling converts raw data into efficient and accurately trained models that can be used for patient risk analysis and medical image processing even when patients exhibit complex comorbidities. Created with BioRender.com.

Levels of low-density lipoprotein (LDL), apolipoprotein B (ApoB), HDL, apolipoprotein A1 (ApoA1), and C-reactive protein (CRP) are significant biomarkers of vascular inflammation and are critical to almost all inflammatory disease processes including atherosclerosis.^14^ Yet inefficiencies in biomarker identification, prioritization, and verification complicate the discovery of new biomarkers for practically all diseases.^15^ Traditional approaches attempt to analyze the correlation between a single variable and disease, thus having a narrow approach. This siloed approach has been exhausted in the past, partially to distinguish biological mechanisms and because only a single disease-related biomarker could be measured in plasma relatively easily. However, with an increase in biological metrics and analytes being sampled, bigger and better-curated datasets across not only patients but across scales are becoming available. These data typically include patient history, tissue, and physiological readouts, cell subsets and percentages, and molecular information such as the metabolism of these cells. Machine learning modeling and analysis of such datasets can provide accurate and clinically practical tools for diagnostics and may prove to be very effective for the discovery of dynamic proteomic and metabolic biomarkers in the field of translational medicine (Fig. 1—machine learning modeling can easily convert raw data into deployable models).

Multiple articles have focused on ML for the role of image processing. For example, Alberto *et al.* reported a comparison of the many methods for characterizing plaque components, plaque morphology, and arterial wall measurements using optical coherence tomography (OCT).^16^ Nils *et al.* also discussed the application of ML in coronary artery disease using cardiac computed tomography (CT) images for the detection and characterization of atherosclerotic plaque.^17^ An important point is that data extracted from the analysis of images as well as the image itself can be utilized by ML to discover novel relationships.

### Recent applications of machine learning analysis in atherosclerosis research

Beyond clinical risk assessment, the scientific community has recognized the benefit of mechanistic discovery and prioritizing biomarkers for diseases. As a result, there has been significant work in interpreting ML approaches for ranking variables focused on finding ways to replace the relevance scores with measures that can be interpreted using standard methods. To this end, a prior study concluded that ML methods, while computationally expensive, are capable of identifying truly relevant biomarkers within datasets with multivariate interacting variables,^18^ helping clear the path for ML analysis of healthcare data (Table 1).

**TABLE I.** Recent applications of machine learning methods in understanding atherosclerosis.

References	Focus	Significant discovery
Ambale-Venkatesh *et al.*^19^	Event prediction	Machine learning methods applied to deep phenotyped datasets showed high predictive cardiovascular event accuracy.^19^
Chen *et al.*^5^	Event prediction	Machine learning methods can be applied to effectively predict risk in patients with severe dilated cardiomyopathy.^5^
Han *et al.*^20^	Risk stratification/feature selection	Machine learning approaches can be used to identify important features related to quantitative atherosclerosis characterization and patients at risk of rapid coronary plaque progression.^20^
Hu *et al.*^21^	Risk stratification/feature selection in comorbidity	Machine learning methods can be used to effectively predict prediabetes at risk for rapid atherosclerosis progression.^21^
Huynh-Thu *et al.*^18^	Validate machine learning for biomarker discovery	Machine learning approaches leveraged to explore methods of features extraction.^18^
Motwani *et al.*^22^	Event prediction	Machine learning applied to clinical and Coronary CT data predicted 5-year all-cause mortality better than using clinical or Coronary CT data alone.^22^
Munger *et al.*^6^	Regression/biomarker discovery	Machine learning methods can be effective in identifying the top predictors of noncalcified coronary burden in psoriasis.^6^
Quesada *et al.*^23^	Risk stratification	Machine learning methods demonstrated better predictive capacity for cardiovascular events and better classification indicators than some traditional risk scores.^23^
Terrada *et al.*^24^	Diagnostic classification	Use of machine learning to develop highly accurate approach toward facilitating large-scale clinical diagnostics for atherosclerosis.^24^
van Rosendael *et al.*^25^	Risk stratification	Risk scoring using machine learning analysis of standard 16 coronary segment stenosis and composition information has better prognostic accuracy than current Coronary CT integrated risk scores.^25^
Weng *et al.*^26^	Risk stratification	Machine learning shows greatly improved accuracy of cardiovascular risk prediction.^26^
Xie *et al.*^27^	Clustering/regression	Weighted machine learning approaches can improve the accuracy of cardiovascular diseases disease risk.^27^

### Machine learning analysis for risk stratification

Studies have shown that ML methods may be better for the prediction of cardiovascular events than traditional risk assessment scales commonly used in clinical practice.^23,26,27^ In 2017, researchers studied the ability of ML techniques to classify six cardiovascular outcomes and compared these results with standard cardiovascular risk scores.^19^ Using the MESA (Multi-Ethnic Study of Atherosclerosis) dataset that included 735 variables from imaging and noninvasive tests, questionnaires, and biomarker panels, they applied a random survival forest method to rank the top-20 predictors of each outcome.^19^ Interestingly, imaging, electrocardiography, and serum biomarkers were shown to be more represented in the top-20 important variables selected when compared to traditional cardiovascular risk factors in predicting cardiovascular outcomes.^19^

More recently, ML approaches are being applied to develop predictive models to classify individuals who are likely to be diagnosed with diseases as a result of atherosclerosis.^24^ Terrada *et al.* introduce a Medical Diagnosis Support Systems (MDSS) after having achieved an accuracy of 98%.^24^ In development, the MDSS was assessed using seven different classification algorithms: Artificial Neural Network (ANN), K-Nearest Neighbor (KNN), Support Vector Machine (SVM), Decision Tree (DT), Naïve Bayes (NB), Classification Ensemble (CE), and Discriminant Analysis (DA).^24^ The MDSS used data from 835 patient medical records who suffer from atherosclerosis, usually caused by coronary artery diseases (CAD), collected from three databases: the Cleveland Heart Disease, Hungarian, and Z-Alizadeh Sani databases. ^24^ The system's input was a combination of variables included in the final dataset and resulted in an accuracy rating of 98%, far more accurate than other approaches.^24^

While such models represent a significant step forward in large-scale computational clinical diagnostics for atherosclerotic diseases, the results highlight the potential of ML as an approach to clinical analysis as well as some areas where more work is needed. A system such as the MDSS need not be primarily focused on any one disease. Indeed, systems such as this can be developed to efficiently classify and diagnose individuals for many different diseases. The biggest challenge in the development of such systems is proper feature selection.^24^ While Terrada *et al.* did achieve a significant accuracy, the widespread deployment of the system across other diseases is limited by the use of domain knowledge feature selection from datasets and feature engineering. The system generated the appropriate features based on external feature engineering because the authors had yet to develop a technique to automate feature selection.^24^

### Machine learning analysis for the characterization of atherosclerotic plaque formation

Imaging methods have significantly advanced in recent decades, allowing for early detection and characterization of atherosclerosis.^28^ Some studies have used ML for the analysis of imaging data to characterize plaque morphology. A more recent study compared the image segmentation accuracy of three popular ML algorithms, convolutional neural networks (CNNs), random forest (RF), and support vector machine (SVM).^29^ Based on each algorithm's ability to characterize the very thin second layer of coronary artery (media), the CNN was shown to be very effective when applied as a feature extractor and RF proved to be a very accurate classifier. The system resulted in an overall classification rate up to 96% when applied for the recognition and specification of the media intima from other tissues.^29^ Of note is the comparison of both traditional ML methods and deep learning. As one of the first applications of a deep learning CNN to coronary artery image segmentation, this offered the opportunity to expand available training datasets. This is because images selected to be manually curated would only require that a small (8 × 8) pixel patch be manually curated. Once the CNN learns the features over this small patch from within the larger image, it can then apply this learned 8 × 8 pixel feature detector to the rest of the image. Thus, more images can be quickly curated and added to the training set.

Moreover, being able to clinically distinguish plaque phenotypes macroscopically is critical as it is known there are certain plaques more vulnerable to rupture.^30^ Molecularly, vulnerable plaques are characterized by an intact thin fibrous cap, necrotic core, neovascularization, and a reduced volume of smooth muscle cells (SMCs).^31^ Macrophages in the fibrous cap release matrix metalloproteinases (MMPs), which, along with high shear stress, promote weakening of the cap.^31^ Additionally, microcalcification, iron accumulation within the fibrous cap, and macrophage cell death contribute to the weakening of the fibrous cap.^31^ Upon rupture, the contents of the necrotic core come into contact with circulating blood, and the coagulation cascade involving platelets is activated in response to the exposure of lipids and tissue factors, which were present in the necrotic core, leading to a thrombus and thus leading to decreased blood flow and a cardiovascular event.^31^ This is a complex process; however, ML methods could be used to extract valuable feature importance in even complicated clinical processes and complex datasets.^27^

### Intracoronary optical coherence tomography image processing

Intracoronary optical coherence tomography (IOCT) is the application of light-based intracoronary endoscopic imaging along with optical coherence tomography to create cross-sectional images of the artery lumen and wall. Recently, ML guided image processing was applied to analyze IOCT images to generate an automated system to characterize atherosclerotic tissue.^32^ The authors used a supervised ML algorithm on image pixels to classify each pixel based on textural features and used those results to estimate the value of the optical attenuation coefficient.^32^ The images were then manually curated, and it was determined that the proposed method obtained an overall classification accuracy of 81.5%.^32^ While the training and validation dataset was relatively small, their work demonstrated that this application of ML showed potential for clinical use. Additionally, ML-based automated image segmentation and analysis can be used effectively to quantify atherosclerosis plaque deposition.^33^ In this study, ML-based OCT image segmentation was used to highlight the borders along the vessel lumen and identify regions of atherosclerotic plaque.^33^

### Coronary computed tomography angiography image processing

**FIG. 2.**
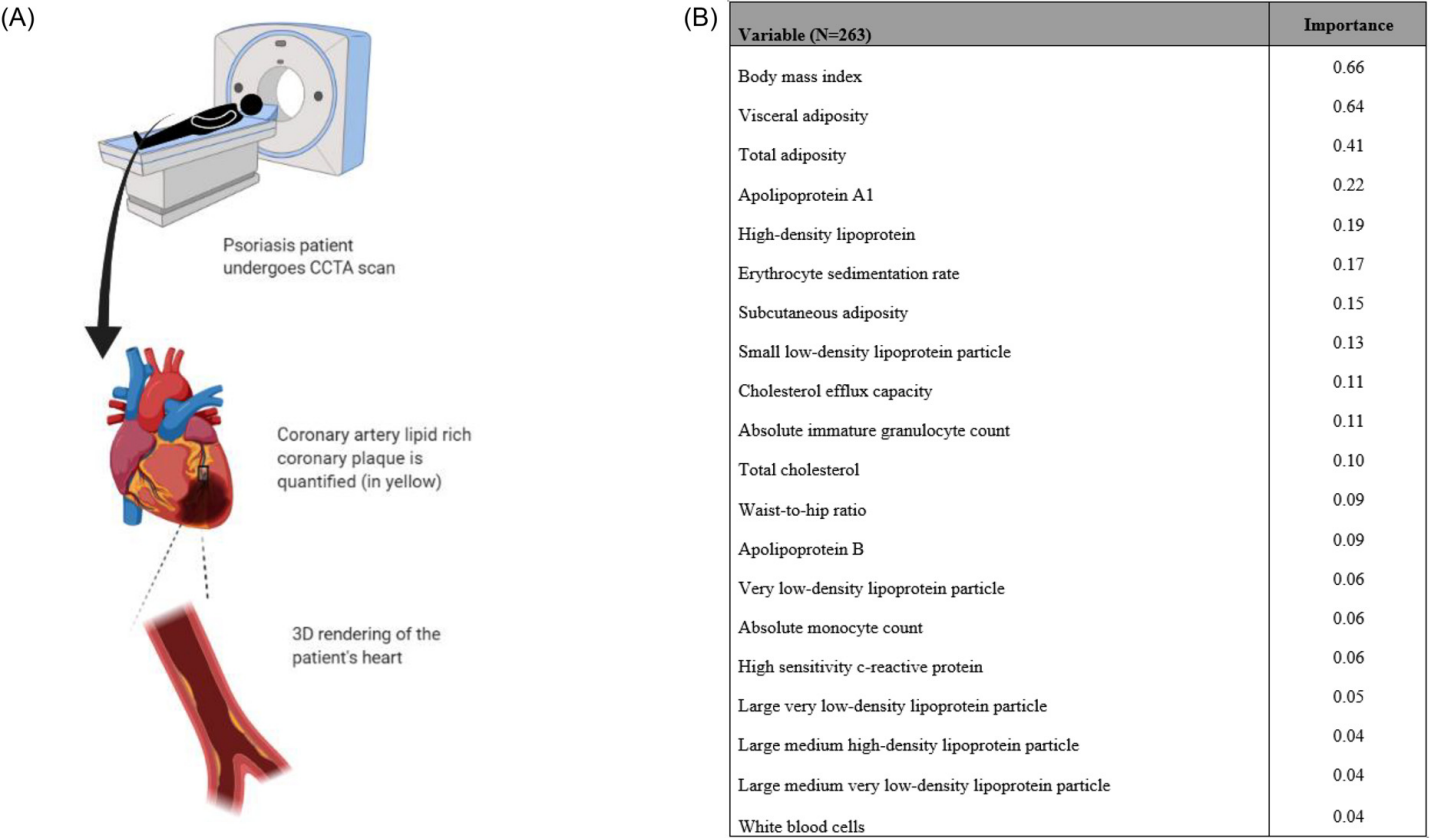
(a) CCTA is used to quantify atherosclerotic plaque in coronary arteries by obtaining a 3D rendering of heart and mapping lipid-rich non-calcified coronary plaque (in yellow). (b) The Top predictors of non-calcified coronary artery plaque burden are generated using ML.

Coronary computed tomography angiography (CCTA) is a noninvasive imaging technique that leverages radiolabeled compounds using computed tomography (CT) to depict the structure and content of the blood vessels including atherosclerotic plaque (Fig. 2). CCTA has proven an effective method for the characterization of coronary plaque burden because CCTA provides a characterization of not only lumen stenosis and arterial remodeling but also plaque subcomponents, including calcified, non-calcified, and high risk features.^30^ A recent ML application related to CCTA was based on data from the PARADIGM (Progression of Atherosclerotic Plaque Determined by Computed Tomographic Angiography Imaging) registry.^20^ The PARADIGM data were collected between 2003 and 2015 as part of a multi-center effort. At the time of the analysis, 1083 consecutive patients had undergone serial CCTA and met the inclusion criteria for the analysis.^20^ After analysis using ML focused on selecting and ranking each feature's importance for classifying individuals at risk for rapid plaque progression, the authors were able to determine that quantitative atherosclerotic plaque characterization was most influential. This was followed by qualitative plaque characterization by CCTA variables and then clinical and laboratory measures.^20^

Often, studies incorporating CCTA also collect several non-imaging-based circulating biomarkers and clinical data as well. In one of the first large scale implementations of ML with CCTA data, it was shown that ML algorithms integrating clinical factors as well as coronary computed tomography angiography (CCTA) data demonstrated superior prediction of 5-year all-cause mortality than clinical or CCTA data alone.^22^ Another study demonstrated that a risk score developed by using ML algorithms had greater prognostic accuracy for cardiovascular disease risk stratification from CCTA readings than the standard CCTA risk scores.^25^

### Application of machine learning in a human disease model of inflammation

Inflammation is critical to the progression of atherosclerosis. Psoriasis, a chronic inflammatory skin disease that affects nearly 2%–3% of the global population, is associated with accelerated rates of atherosclerosis, especially non-calcified coronary plaques.^34,35^ It is now well known that atherosclerotic plaque deposition is accelerated by untreated chronic inflammation in coronary arteries.^36^ One recent application of ML used the Psoriasis Atherosclerosis Cardiometabolic Initiative (PACI) dataset to investigate biomarkers associated with this elevated cardiovascular risk in psoriasis. A combination of feature engineering and the application of ML methods has been used to demonstrate the mapping of complex biological data to a specific clinical outcome and that ML methods can be leveraged to identify top predictors of non-calcified coronary burden in patients with psoriasis.^6^ The analysis showed that the top-20 clinically significant biomarkers were body mass index, visceral adiposity, total adiposity, apolipoprotein A1, high-density lipoprotein, erythrocyte sedimentation rate, subcutaneous adiposity, small low-density lipoprotein particle, cholesterol efflux capacity, absolute granulocyte count, total cholesterol, waist-to-hip ratio, apolipoprotein B, very-low-density lipoprotein particle, absolute monocyte count, high-sensitivity C-reactive protein (hs-CRP), large very-low-density lipoprotein particle, large medium high-density lipoprotein particle, large medium very-low-density lipoprotein particle, and white blood cells (Fig. 2—use of coronary computed tomography angiography in psoriasis).^6^ These biomarkers are known to be related to obesity, dyslipidemia, and inflammation, which are relevant to the progression of atherosclerosis in this unique cohort.^6^ Particularly, in psoriasis patients, systemic inflammation is believed to affect a change in the inner lining of arteries causing an expression of adhesion molecules by endothelial cells. This change increases the capture and translocation of leukocytes into the intima.^37^ Additionally, high-density lipoprotein (HDL) is converted to oxidized HDL in inflammatory states such as psoriasis, thus losing its protective effect.^38^

This conversion decreases the protective function of HDL, leading to a more atherogenic profile and decreased cholesterol efflux ability, all of which promote plaque formation.^38^ Indeed, patients with psoriasis are at increased risk of suffering a cardiovascular event compared with the general population, presumably due to accelerated atherogenesis.^39^ One study demonstrated that severe psoriasis confers an additional 6.2% absolute risk of a 10-year rate of a major cardiovascular event.^40^

**FIG. 3.**
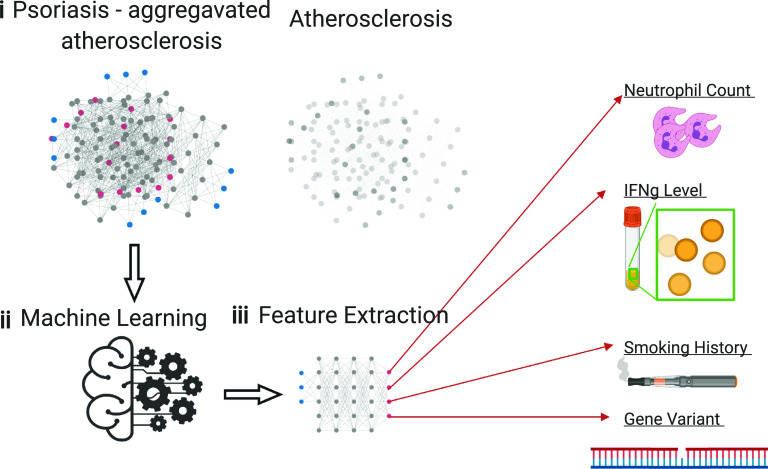
(i) Psoriasis offers an extenuated view into atherosclerosis. (ii) The biologically meaningful parameters' signals will be amplified and easier to analyze with machine learning. (iii) This will enable significant extraction of potential biomarkers that could be used concomitantly and also explored for a biological mechanism for atherosclerosis so that they can be used as druggable targets. Created with BioRender.com.

This application of ML to atherosclerosis, within the psoriasis group, gives us a unique perspective and window into studying atherosclerosis since people suffering from inflammatory diseases such as psoriasis have a high prevalence of cardiovascular disease. Because of the extenuated inflammation, the progression of the disease, altered molecular networks, disrupted cellular communication, and aggravated skin disease pathophysiology in psoriasis, this biological amplification of biomarkers seen in psoriasis could be used to understand the complex process of atherosclerosis. These boosted biological signals from a condition that magnifies atherosclerotic disease could increase the signal to noise ratio to elucidate the key players and their relationships amongst the many biological players and processes known (Fig. 3—concept of feature extraction from raw data).

### Future directions and conclusions

Cumulatively, our review demonstrates that valuable insights can be extracted when ML is applied to clinical datasets. As a direct result of the recent application of ML in atherosclerosis, the number of known molecular, cellular, and physiological factors affecting atherosclerosis has dramatically increased. Most of these factors have been studied individually, leaving questions as to how many of these factors are interrelated and correlated. Adding environmental factors and broad lifestyle choices to this type of analysis could be particularly interesting from an interventional public health point of view. Thus, ML analysis of broad, well-characterized datasets will lead to a better understanding of how such factors are interconnected and impact cardiovascular health.

Furthermore, given that studies show the physiological pathways involved in atherogenesis are shared by other diseases, studying these diseases in tandem could be fruitful. A significant complication of atherosclerosis is the fact that there are a few symptoms until a cardiovascular event occurs.^41^ We imagine a deeper understanding of the condition could be achieved by using ML to analyze a targeted, well-characterized dataset of many variables important to atherosclerosis in combination with other conditions/comorbidities that may serve as surrogates for gauging the severity of underlying atherosclerosis.

We expect four major areas of advancement from applying ML to atherosclerosis. First, being able to identify many salient variables will establish new biomarkers that could be used clinically. In a recent evaluation of the feasibility of creating a simple and clinically useful diagnostic panel for heart failure with reduced ejection fraction, the authors took a combined approach using untargeted metabolomics and ML.^42^ It was concluded that the combination of untargeted metabolomics and ML algorithms was a promising tool for the diagnostic workup in heart failure with reduced ejection fraction.^42^ However, large scale, multi-center studies of the methods are crucial for conformation and clinical utility of these tools.

Second, the ability to look at many variables simultaneously may inform us of the actual biological mechanisms and interactions across many different levels of biology from molecules to tissues. It is known that biomarkers play a significant role in the management, diagnosis, prognostication, and screening of diseases such as atherosclerosis. However, the utility of novel, emerging biomarkers is less established. Machine learning analysis allows for a multimodal approach that can simultaneously include many biomarkers, which can help identify multiple different pathophysiological pathways. This would provide a more integrated and informed assessment of the state of the patient, which would provide an improved measurement of risk compared to traditional risk scores.^43^ In a recent clinical trial focusing on the treatment of preserved cardiac function heart failure patients with an aldosterone antagonist, 49 plasma biomarkers were measured in 379 trial participants.^44^ The aim was to understand the relationship between these biomarkers and the risk of all-cause death or heart failure-related hospital admission. It was concluded that a multimarker ML approach was a promising strategy for accurate risk stratification.^44^

Third, this may increase our current understanding of the relationship of multiple diseases and disease states, allowing a better understanding of complex “multimorbidity” and opening the opportunity for common interventions. Understanding complex multimorbidity is a significant challenge, and as a result, many diseases are studied in isolation. Machine learning provides tools that can be applied to overcome the many challenges in multimorbidity, but only a small percentage have been used for the study of multimorbidity.^45^ Hu *et al.* demonstrated that ML can effectively predict prediabetics at risk for rapid atherosclerosis progression.^21^

Finally, the information these studies provide will highlight the pathway of collecting the next set of datasets for further iterations of ML discoveries. Machine learning techniques are capable of identifying clinically relevant patterns hidden amongst an abundance of potentially irrelevant information.^46^ In a recent review of the application of ML in autoimmune disorders, the authors established that novel ML methods were being applied to data on multiple sclerosis, rheumatoid arthritis, and inflammatory bowel disease. Datasets used often included different data types in the modeling process.^47^ This work shows that complex predictive models may be improved through the integration of specific data types and that an understanding of the best data to include in such models may not be the same as the data traditionally collected.

One limitation in the application of ML in current datasets is that the prospective follow-up data are largely incomplete. We feel that these prospective data will permit longitudinal analyses across phenotypes of interest, thereby accelerating understanding of potential biologic pathways. This has made predicting the value of surrogate endpoints of disease states very difficult. To solve this problem, researchers will need to continue to fill and expand datasets from prospective studies while learning to adapt many of the traditionally reliable ML algorithms to evaluate high dimensional time-series data. Another limitation is that cross-validation using separate testing and validation datasets for robust model evaluation is not always an option because, while many of the best practices of validation, cross-validation, and independent testing of ML models have been discovered, very few well-characterized datasets related to atherosclerosis are available. Finally, it is well known that the appropriate application of these methods in solving complex problems in the domain of natural language processing or image and speech recognition has been extremely successful. We and others have successfully applied these methods on available high-quality datasets to highlight complicated relationships between multiple diseases. With continued increases in computing power and the vast expansion of well-characterized time-series biological and clinical data expected to be available very soon, now is the time for the application of ML methods to subclinical and interrelated diseases. In conclusion, here we provide a review of how ML can accelerate our understanding of atherosclerosis and some of the key applications of ML to these datasets for characterizing novel biomarkers of atherosclerotic disease progression. Much larger studies on well-characterized datasets will be possible as technology and analytical understanding improve in the future.

## Data Availability

No human data or animal data are included in this review and thus data sharing agreement does not apply to this article.
